# An improved method for inducing prometaphase chromosomes in plants

**DOI:** 10.1186/s13039-018-0380-6

**Published:** 2018-05-10

**Authors:** Agus Budi Setiawan, Chee How Teo, Shinji Kikuchi, Hidenori Sassa, Takato Koba

**Affiliations:** 1 Laboratory of Genetics and Plant Breeding, Graduate School of Horticulture, Chiba University, Matsudo, Chiba 271-8510 Japan; 2 Center for Research in Biotechnology for Agriculture, University of Malaya, 50603 Kuala Lumpur, Malaysia

**Keywords:** Prometaphase, Pachytene, Chloroform, FISH, *Cucumis melo*, *Abelia* × *grandiflora*

## Abstract

**Background:**

Detailed karyotyping using metaphase chromosomes in melon (*Cucumis melo* L.) remains a challenge because of their small chromosome sizes and poor stainability. Prometaphase chromosomes, which are two times longer and loosely condensed, provide a significantly better resolution for fluorescence in situ hybridization (FISH) than metaphase chromosomes. However, suitable method for acquiring prometaphase chromosomes in melon have been poorly investigated.

**Results:**

In this study, a modified Carnoy’s solution II (MC II) [6:3:1 (*v*/v) ethanol: acetic acid: chloroform] was used as a pretreatment solution to obtain prometaphase chromosomes. We demonstrated that the prometaphase chromosomes obtained using the MC II method are excellent for karyotyping and FISH analysis. We also observed that a combination of MC II and the modified air dry (ADI) method provides a satisfactory meiotic pachytene chromosome preparation with reduced cytoplasmic background and clear chromatin spreads. Moreover, we demonstrated that pachytene and prometaphase chromosomes of melon and *Abelia* × *grandiflora* generate significantly better FISH images when prepared using the method described. We confirmed, for the first time, that *Abelia* × *grandiflora* has pairs of both strong and weak 45S ribosomal DNA signals on the short arms of their metaphase chromosomes.

**Conclusion:**

The MC II and ADI method are simple and effective for acquiring prometaphase and pachytene chromosomes with reduced cytoplasm background in plants. Our methods provide high-resolution FISH images that can help accelerate molecular cytogenetic research in plants.

## Background

Chromosome preparation is crucial for cytogenetic studies. Fluorescence in situ hybridization (FISH), a molecular cytogenetic technique, requires properly dispersed metaphase or prometaphase chromosomes for its application. Melon (*Cucumis melo* L.) belongs to the *Cucurbitaceae* family and is a diploid species having 2*n* = 2*x* = 24 chromosomes [1]. Detailed karyotype analysis in the *Cucumis* genus, particularly in melon, has been difficult to achieve because of their small chromosome sizes and poor stainability [2, 3]. In addition, the identification of secondary constrictions and the procurement of more detailed chromatin images are also difficult, even when using properly dispersed metaphase chromosomes, because of their highly condensed status. For these reasons, we propose the use of prometaphase chromosomes for FISH analyses in melon. Prometaphase chromosomes are effective and preferable for cytogenetic analyses and identification of individual chromosomes because the chromosomes are easily distinguishable due to the uneven condensation of chromatin fibers along chromosomes [4]. FISH using prometaphase chromosomes has been successfully applied in studies involving *Brassica* [5], rice [6–8], *Catharanthus roseus* [9], and *Lablab purpureus* [10]. However, suitable methods to induce prometaphase chromosomes in other plants have been poorly investigated.

Prometaphase chromosomes in *Brassica* [5] and rice [7] have been successfully induced using ethanol and acetic acid (3:1) without pretreatment. Other methods to accumulate metaphase and prometaphase chromosomes, such as with ice water (ice) treatment for 24 h [11] or 0.002 M 8-hydroxyquinoline (8-Hq) [12, 13] have also been reported. Although FISH studies have also been reported in melon [12–15], most of them used metaphase chromosomes, which are shorter and more compact than prometaphase chromosomes.

Modified Carnoy’s solution II (MC II) made up of ethanol: acetic acid: chloroform (6:3:1) has been previously used to produce super-stretched pachytene chromosomes in maize [16]. However, its efficiency in inducing prometaphase chromosomes in mitotic cells in other plant species has not yet been reported. Here, using melon, we compared the effectiveness of MC II with other methods including Carnoy’s solution (ethanol: acetic acid (3:1)), 24-h ice water treatment and 0.002 M 8-Hq for the induction of properly dispersed prometaphase and metaphase chromosomes. We demonstrated that MC II can be used for FISH analysis with enhanced chromosome distribution in melon. Furthermore, we provide a protocol for obtaining pachytene chromosomes from melon and *Abelia* × *grandiflora* flower buds without the need to squash the slides. This is achieved by a combination of MC II and the modified air dry (ADI) methods.

## Methods

### Plant materials

*Cucumis melo* L. subsp. *melo* var. cantalupo Ser. cultivar ‘Baladewa’ (local name, Blewah), a commercial melon in Indonesia, was used in this study for FISH analyses. *Abelia* × *grandiflora*, a hybrid plant between *Abelia chinensis* and *Abelia uniflora*, was also used to test the effectiveness of MC II and ADI methods in obtaining pachytene chromosomes. The plant was grown and maintained at the Graduate School of Horticulture, Chiba University, Matsudo, Japan.

### Experimental design

The present experiment was performed using a completely randomized design (CRD) with four treatments as follows: (1) Melon root tips from germinated seeds were treated with the MC II method [16]. In this method, the root tips were pretreated with freshly prepared 6:3:1 (v/v) ethanol: acetic acid: chloroform for 3–4 h at room temperature (RT), and then fixed in 3:1 (v/v) ethanol: acetic acid solution (C3:1) for 5 days at 4 °C. (2) Root tips were pre-treated with 0.002 M 8-Hq for 4 h at RT and then fixed in C3:1 (v/v) for 5 days at 4 °C. (3) Root tips were pretreated with Ice for 24 h and then fixed in C3:1 for 5 days at 4 °C. (4) Root tips were directly fixed in C3:1 for 5 days at 4 °C. Each treatment was replicated three times using 10 root tips per replication. Three slides of each replication from each treatment were chosen for chromosome data analysis.

### Mitotic chromosome preparations

The seeds were germinated on moistened filter paper kept in petri dishes in a growth chamber at 25 °C. The main root tips (0.5–1 cm) were cut, and the germinated seeds were transplanted into potting trays maintained in a greenhouse. Additional root tips could be harvested from the transplanted melon plants. The root tips of each treatment were washed in 1 ml of enzyme buffer (40 ml of 100 mM citric acid + 60 ml of 100 mM sodium citrate, pH 4.8) for 10 min [11]. The meristematic root tips were cut and macerated in 15 μl of enzyme mixture containing 4% Cellulose Onozuka RS (Yakult), 2% Pectinase (Sigma), and 1% Pectolyase Y-23 (Kyowa Chemical, Osaka, Japan) at 37 °C for 1 h. The enzyme mixture was cleaned off from the meristematic root tips using Kimwipe tissues. Then, 10 μl of 60% acetic acid was added onto the root tips and left until the root tips became transparent or pale, and an 18 × 18 mm cover slip was placed over the slide. The slides were tapped using probe needles to spread the cells, squashed, and flame-dried over an alcohol flame for a few seconds. Finally, the slides were kept at − 81 °C for 1–2 days, and the cover slips were removed before FISH.

### Meiotic chromosome preparations

We selected MC II for pachytene chromosome preparation as we found it to be preferable for inducing prometaphase and metaphase in melon cells when combined together with the ADI method [17]. The melon and *Abelia* × *grandiflora* flower buds were pretreated with 6:3:1 (v/v) ethanol: acetic acid: chloroform for 3–4 h at RT and fixed in C3:1 for 5 days at 4 °C. The flower buds were washed in 1 ml of enzyme buffer (40 ml of 100 mM citric acid + 60 ml of 100 mM sodium citrate, pH 4.8) for 10 min. The anthers were dissected using the forceps under a stereomicroscope and macerated in 15 μl of the enzyme mixture described above at 37 °C for 1 h. The enzyme mixture surrounding the meristematic anthers was cleaned using Kimwipe tissues. The ADI method consisted of three main solutions, namely, fixative I [60% of 1:1 (*v*/v) acetic acid:ethanol in distilled water; 3 ml of glacial acetic acid + 3 ml ethanol (> 99.5%) + 4 ml distilled water], fixative II [4 ml absolute 1:1 (v/v) acetic acid: ethanol; 2 ml glacial acetic acid + 2 ml ethanol (> 99.5%)], and fixative III (2 ml glacial acetic acid). Fixatives I and II were freshly prepared before use; Fixative I should not be stored overnight because it degrades rapidly into ethyl acetate, which is detrimental to cells [17]. Fixative I was applied (5 μl) to the anthers, and the anthers were rapidly macerated using forceps to spread the pollen mother cells (PMCs). Fixative I was applied once again (5 μl), and most of it spread out to the edge of the slide. After 1–2 min, fixative I had evaporated. To avoid drying of the slide, 5 μl of fixative II was quickly applied and left to spread to the edge of the slide. Finally, 5 μl of fixative III was applied, and the slide was air dried. The excess fixative on the edge of the slide was removed using filter paper. The slides can be stored in a slide box at RT up to 6 months without noticeable degradation.

### Probe preparations

Wheat 45S ribosomal DNA (rDNA; pTa71) and melon centromere satellite DNA (Cmcent) were used as the probes [18, 19]. The Cmcent probe was labeled with the dig-nick translation mix (Roche) or biotin-nick translation mix (Roche), while the 45S rDNA probe was labeled with the dig-nick translation mix (Roche).

### FISH analysis

Slides containing melon chromosomes were pretreated with 200 μl RNase A solution per slide [2 μl of 10 mg/ml RNase A + 20 μl of 20× saline sodium citrate (SSC) + 178 μl of sterile distilled water (SDW)], incubated at 37 °C for 1 h, washed in 2× SSC for 2 min and air dried. The slides were further pretreated with 150 μl of pepsin solution per slide (1.5 μl of 500 μg/ml pepsin + 0.25 μl of 6 N hydrochloride acid + 148.25 μl of SDW), incubated at 37 °C for 30 min, washed in 2× SSC for 2 min and air dried. The slides were re-fixed with 100 μl of 1% paraformaldehyde per slide for 10 min at RT, washed in 2× SSC for 2 min and air dried. The slides were hybridized with 10 μl of hybridization cocktail (5 μl of formamide + 2 μl of 50% dextran sulfate + 1 μl of 20× SSC + 1–2 μl probe) per slide and covered with a cover slip (22 × 22 mm). The edges of the cover slips were sealed with rubber cement and denatured on a hot plate at 80 °C for 2–3 min. Finally, the slides were placed in a humidity chamber and incubated at 37 °C overnight. Following hybridization, the slides were washed in 2× SSC for 2 min to remove the cover slips, washed in milliQ water for 1 min, and air dried. Following this, 126 μl detection solution [125 μl of 1% BSA in 4× SSC + 0.5 μl of 0.4 μg/ml anti-digoxigenin rhodamine (Roche) + 0.5 μl of 0.5 μg/ml biotinylated streptavidin-FITC (vector laboratories)] was applied for detection and the slides were incubated at 37 °C for 30 min. The slides were then washed in 2× SSC for 2 min, in milliQ water for 1 min, and air dried. Finally, the slides were counter-stained using 5 μg/ml 4,6-diamidino-2-phenylindole (DAPI) in a VectaShield antifade solution (Vector Laboratories). The slides were observed under a fluorescence microscope (Olympus BX53) equipped with a cooled CCD camera (Photometrics CoolSNAP MYO), processed by Metamorph, Metavue imaging series version 7.8 and edited with Adobe Photoshop CS 6.

### Data analysis

Total chromosome length and percentage of cells showing properly dispersed chromosomes per slide were measured for each treatment. Total chromosome length was measured in 20 properly dispersed cells. The total number of cells having properly dispersed chromosomes per slide was determined by tracing the entire slide using 40× magnification. The data were analyzed using analysis of variance according to CRD and Duncan’s multiple range test. The statistical analyses were performed using SAS 9.3 software package (SAS Institute, USA).

## Results

### Modified Carnoy’s solution II increases the prometaphase index

**Fig. 1:**
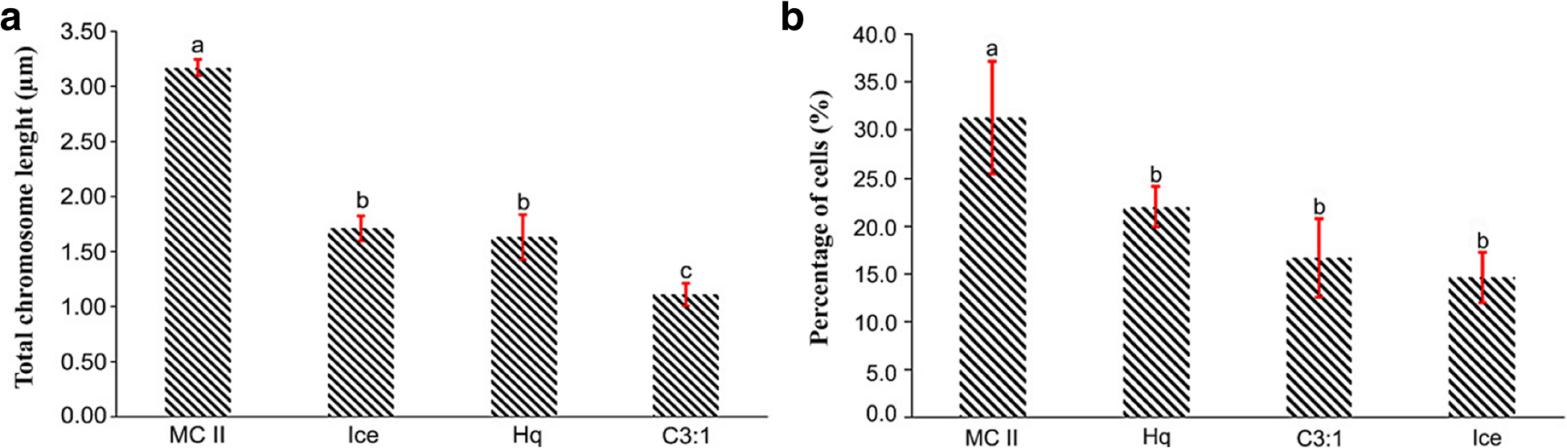
The total chromosome length (**a**) and percentage of cells showing properly dispersed chromosomes per slide (**b**) that were obtained by four methods, i.e., MC II, 8-Hq, ice, and C3:1. Note: bars with the same letters are not significantly different at *P* ≤ 0.05. Red lines depict standard deviations

Four methods, namely, MC II, 0.002 M 8-Hq for 4 h, ice treatment for 24 h, and C3:1 were compared for their effectiveness in obtaining properly dispersed prometaphase and metaphase chromosomes in melon. The root tips were harvested between 7 am and 9 am and treated as mentioned above. The total chromosome lengths obtained using MC II (3.17 μm) were significantly longer than those obtained using the other three methods (1.1–1.7 μm, Fig. 1a). This result suggests that melon chromosomes obtained using MC II are twice as longer as those with the other methods. In addition, the total number of cells having properly dispersed chromosomes per slide using MC II was significantly higher than those obtained using the 8-Hq, ice, and C3:1 methods (Fig. 1b). Thus, these results suggest that the MC II method is effective for increasing the prometaphase index in pretreated root tips. Cells with numerous chromosomes that are properly dispersed are essential for FISH in order to properly visualize the chromosomes.

### Prometaphase chromosomes offer details on melon chromosome morphology

**Fig. 2:**
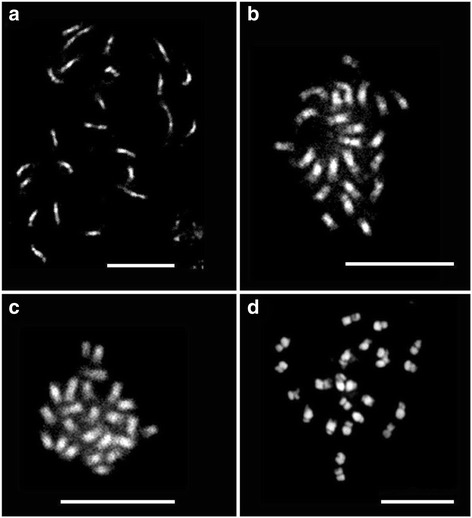
Melon chromosomes obtained by the four treatments: prometaphase chromosomes using the MC II method (**a**), metaphase chromosomes using the ice treatment (**b**), metaphase chromosomes using the C3:1 treatment (**c**), and metaphase chromosomes using the 8-Hq treatment (**d**). Scale bars = 10 μm

Prometaphase chromosomes were successfully arrested in melon by the MC II method (Fig. 2a). These chromosomes were larger and less condensed than the metaphase chromosomes obtained using the 8-Hq, ice, and C3:1 methods (Fig. 2b, c, and d). Using MC II-treated prometaphase chromosomes, we were able to clearly detect the heterochromatic and euchromatic regions. Most of the metaphase chromosomes obtained using the 8-Hq, ice, and C3:1 methods had almost the same sizes for each individual chromosome, and no difference was observed in the total chromosome lengths (Fig. 2b, c, and d). In contrast, prometaphase chromosomes treated by the MC II method showed different chromosome lengths for each individual chromosome (Fig. 2a). The ease of identifying individual chromosomes by size, as a result of uneven condensation of each individual prometaphase chromosome, is an advantage of using prometaphase chromosomes for karyotyping [4]. Prometaphase chromosomes with different degrees of condensation can be used for initial karyotyping of a plant in cases where DNA probes are not available to distinguish individual chromosomes, particularly for newly karyotyped plant species with small chromosome sizes.

### Preparation of pachytene chromosomes using a combination of MC II and ADI methods

**Fig. 3:**
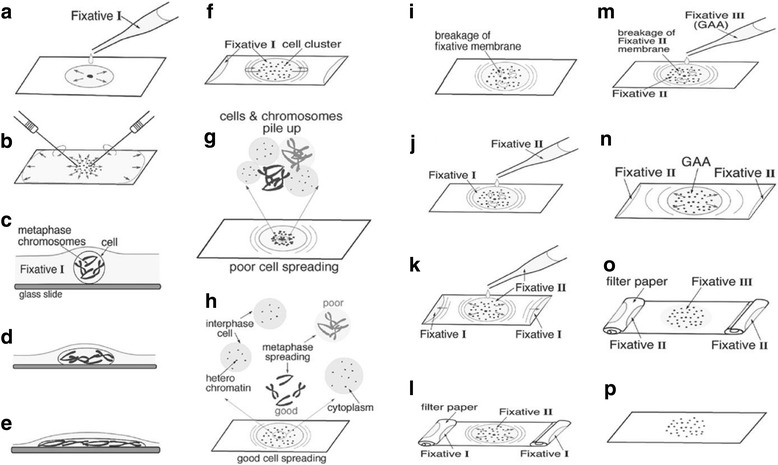
Illustration of the ADI method. The figures are modified with permission [17]. The anthers are treated with enzyme mixtures and incubated at 37 °C for 1–2 h. After incubation, the enzyme surrounding the anther is cleaned using a Kimwipe tissue. Fixative I solution (5-10 μl) is applied directly onto the anther (**a**). The anther is macerated as quickly as possible using dissected needles (**b**). Most of fixative I spreads out to the edge of the slide but partially remains around the cells. After a few minutes, the fixative I evaporates, leaving behind a thin layer, and the spherical cells floating in the fixative adhere to the slide by surface tension (**c-f**). If the humidity is less than 50%, most of the fixative evaporates before the cell becomes flat, and the chromosomes aggregate together (**g**). If the temperature is > 25 °C and humidity is > 80%, the fixative retracts again to the center of the slide (**f**). If the temperature is 20 °C with 65%–70% humidity, the evaporation speed of the fixative and flattening of the cell by surface tension are balanced (**h**). When the breakage of the fixative membrane covers approximately half of the area of the spreading cells (**i**), 5–10 μl of freshly prepared fixative II is added (**j**). Fixative I (60%, 1:1) then moves to the edge of the slide immediately (**k**) and is replaced by fixative II (absolute, 1:1). Fixative II should be applied before the breakage of the fixative membrane to allow expansion of the chromosomes. Fixative I accumulation at the edge of the slide can be removed with rolled filter paper (**l**). When the breakage of the fixative II membrane covers approximately half of the area of the spreading cells, 5–10 μl of freshly prepared fixative III is added (**m**), and fixative II moves to the edge of the slide (**n**). Fixative III is effective in removing cytoplasm and cleaning up the background of metaphase spreads. Fixative II accumulation at the edge of the slide can be removed with rolled filter paper (**o**) to dry the slide completely (**p**)

**Fig. 4:**
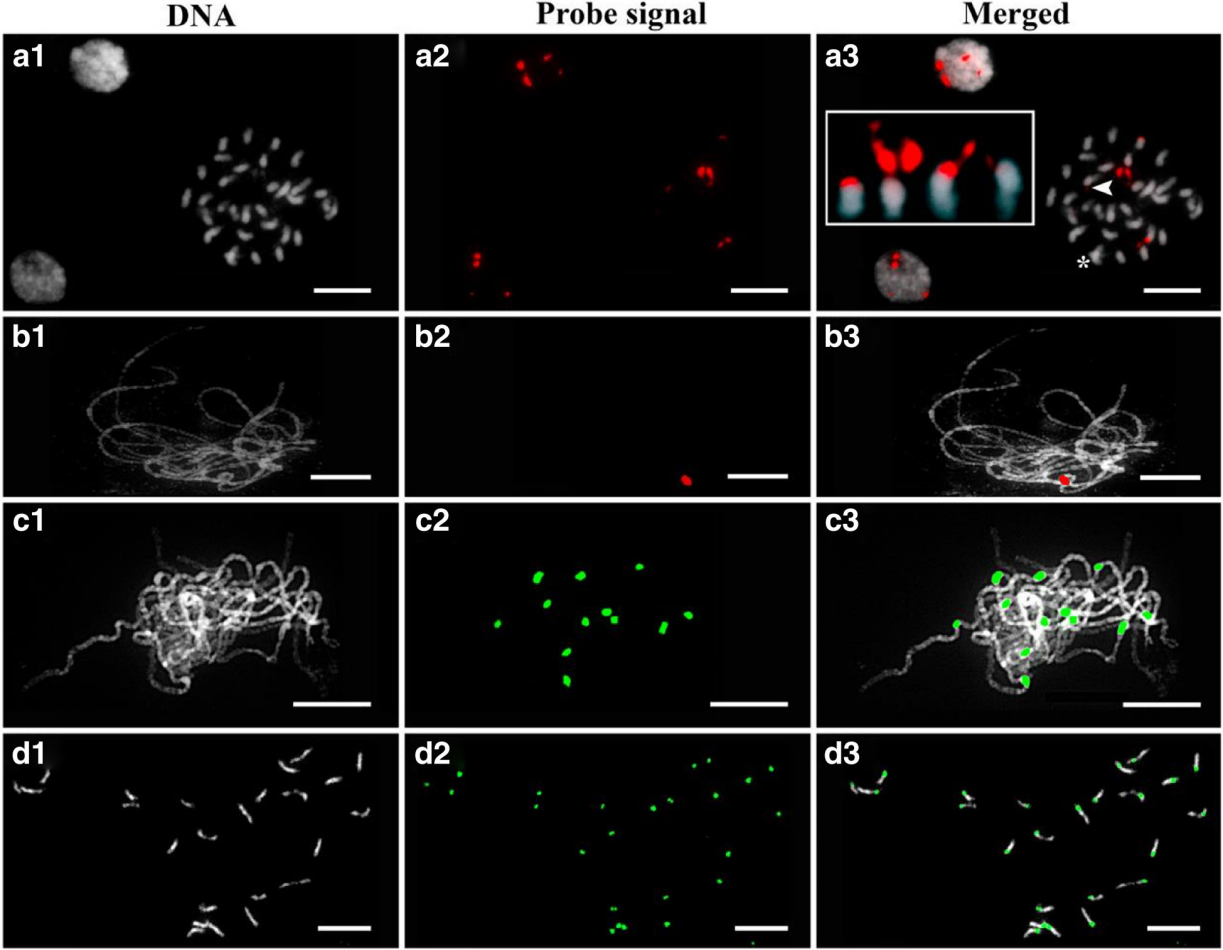
Prometaphase and pachytene chromosomes of *Abelia × grandiflora* (**a** and **b**) and *Cucumis melo* (**c** and **d**). FISH detection of 45S rDNA in somatic metaphase (**a1**-**a3**) and pachytene (**b1**-**b3**) chromosomes of *Abelia × grandiflora*. Arrowhead shows a weak signal of 45S rDNA. Asterisk shows overlapped chromosomes, and inset shows the arrangement of two pairs of chromosomes with 45S rDNA signals (**a3**). FISH detection of melon centromeres in pachytene cells (**c1**-**c3**) and prometaphase cells (**d1**-**d3**). Somatic metaphase and pachytene chromosome of *Abelia × grandiflora* (**a1** and **b1**) and melon pachytene chromosomes (**c1**) were acquired using a combination of MC II and ADI methods. Melon prometaphase chromosome spread (**d1**) was obtained using the germinated root with the MC II method. Red signals in **a2** and **b2** are 45S rDNA. Green signals in **c2** depict Cmcent labeled with digoxigenin. Green signals in **d2** are Cmcent labeled with biotin. Scale bars = 10 μm

The high degree of cytoplasm present in PMCs caused a reduction in FISH resolution and was the primary obstacle for chromosome preparation when using the squash method. The ADI method for karyotyping ant chromosomes was developed by Dr. Hirotami T. Imai [17]. In this experiment we used a combination of the MC II and ADI methods to prepare pachytene chromosome slides from melon and *Abelia* × *grandiflora* flower buds (Figs. 3, 4b1 and c1). These pachytene cells showed reduced cytoplasm and clear chromosomes. We also obtained properly dispersed metaphase II and interphase cells with no cytoplasmic background in *Abelia* × *grandiflora*, and confirmed that it had 32 chromosomes (Fig. 4a1). In this method, freezing the slides at an ultralow temperature is unnecessary, and the slides can be directly used for FISH after being inspected using phase-contrast microscopy.

### FISH analyses of melon and *Abelia* × *grandiflora* chromosomes

Chromosome spreads obtained from root tips and PMCs prepared by the MC II method alone as well as a combination of the MC II and ADI methods, were evaluated for their applicability for FISH analysis of somatic metaphase and meiotic pachytene chromosomes of *Abelia* × *grandiflora* and melon. FISH performed on somatic metaphase and pachytene chromosomes of *Abelia* × *grandiflora* using a 45S rDNA probe revealed four metaphase and two pachytene hybridization signals (Fig. 4a3 and b3). One pair of metaphase chromosomes had strong signals, whereas the rest had weak signals, with both signals being located on the short arms. FISH performed on interphase nuclei also showed four signals of 45S rDNA in *Abelia* × *grandiflora* (Fig. 4a3). These results suggest the conservation of 45S rDNA on two pairs of chromosomes in *Abelia* × *grandiflora*.

Twelve signals of Cmcent located at heterochromatic blocks were detected in melon pachytene chromosomes (Fig. 4c3), similar to previously reported results [14]. Cmcent, a marker associated with the melon centromere, was successfully hybridized to melon prometaphase chromosomes. The signal positions on the chromosomes were consistent with previously described results [18]. In addition, the chromosome morphology prior to the FISH experiment (DAPI images) was preserved even after FISH. Different chromosome morphologies are typically observed during FISH experiments, caused by the effect of heat treatment on chromosomes, inducing changes in chromatin conformation. Another method reported that using the steam-drop method can induce chromatin protrusion and requires a pretreatment before proceeding to FISH [20]. In this study, we demonstrate that prometaphase chromosomes obtained using the MC II method are satisfactory for FISH experiments in plant species.

## Discussion

Chromosome preparation is a key factor in FISH experiments and karyotyping. Every plant species has different chromosome sizes and intracellular components [21] that may affect the choice of chromosome preparation techniques used to obtain a satisfactory chromosome slide. Many researchers use different fixative solutions to arrest metaphase or prometaphase chromosomes, such as 2.5 μM amiprophos-methyl in *Vicia faba* L. [22], 0.002 M 8-Hq in melon [14], and α-bromonaphthalene in *S. formosissima* [21] and orchids [23]. Metaphase chromosomes have been used for karyotyping and FISH in melon [12–14, 18]. Chromosome sizes in melon are small, and the metaphase chromosomes are too condensed for FISH karyotyping analysis. Therefore, we chose prometaphase chromosomes, which are less condensed and display more detail than metaphase chromosomes. Because of uneven condensation patterns, prometaphase chromosomes have been found to be more convenient for karyotyping in plant species with small chromosome sizes [4].

The MC II method was effective in arresting the prometaphase chromosomes in the present study. In addition, using this method, the prometaphase index was increased, i.e., the number of properly dispersed chromosomes per slide and the total length of chromosomes were both significantly increased. The pretreatment was conducted between 7.00 am and 9.00 am at which time the root meristem was actively growing. In contrast to the MC II treatment, results obtained using the 8-Hq, ice, and C3:1 treatments were consistent with those of previous studies, where metaphase chromosomes were mostly arrested as reported in *Humulus japonicus* [20], *Hippeastrum puniceum* [24], *Passiflora* [25], melon [26, 27], *Papaver* sp. [28], and *Triticale* [29]. Therefore, timing of the pretreatment along with MC II treatment was found to be significantly important for arresting most of the cells in prometaphase condition. In addition, the morphologies of prometaphase and pachytene chromosomes before performing FISH (DAPI images) were preserved even after the completion of FISH. This may be due to the combination of ethanol, acetic acid, and chloroform, which prevents the considerable shrinking of tissue with hydrogen bonding that stabilizes and preserves the tissue structures [30].

MC II can be used to prepare super-stretched pachytene chromosomes in maize, that are more robust and durable compared with those acquired by other fixative solutions [16], whereas the ADI method was developed primarily for ant chromosomes [17]. We demonstrated that a method combining the MC II and ADI methods can be used for plant species. We obtained satisfactory pachytene chromosomes with reduced cytoplasm in melon and *Abelia* × *grandiflora*. Furthermore, we reduced the time required for FISH by skipping the slide-freezing step used in the chromosome squash method. The ADI method consists of three main fixative solutions; fixative I is important to spread the chromosomes evenly on the slide, fixative II is critical to obtain satisfactory and properly dispersed metaphase and pachytene chromosomes, and fixative III is used to remove the cytoplasmic background of the metaphase and pachytene chromosome cells [17].

## Conclusions

The results reported here demonstrate the effectiveness of the MC II method for inducing prometaphase chromosomes in plant species. The root tips treated by this method demonstrate an increased prometaphase index. In addition, the prometaphase chromosome distribution prepared with this method reveals more distinct patterns of heterochromatic, euchromatic, and centromere regions than metaphase chromosomes. The combination of the MC II and ADI methods also provides high-resolution pachytene chromosomes with reduced cytoplasmic background. Physical mapping of 45S rDNA and Cmcent was used to test the applicability of chromosome distribution prepared by those methods. The signals of those probes were easily detected and different chromatin conformations were not found after completion of FISH experiment.

## Abbreviations

ADI: Air dry Imai
ANOVA: Analysis of variance
C3:1: 3:1 (v/v) ethanol: acetic acid
DAPI: 4,6-diamidino-2-phenylindole
FISH: Fluorescence in situ hybridization
HCl: Hydrochloride acid
Hq: 8-hydroxyquinoline
Ice: Ice water
MC II: Modified Carnoy’s solution II
PMC: Pollen mother cell
rDNA: Ribosomal DNA
RT: Room temperature
SDW: Sterile distilled water
SSC: Saline sodium citrate
